# Orbitropic Effect in Superfluid ^3^He B-phase Boundaries

**DOI:** 10.1038/s41598-018-31407-4

**Published:** 2018-09-18

**Authors:** Manuel Arrayás, Richard P. Haley, George R. Pickett, Dmitry Zmeev

**Affiliations:** 1 Área de Electromagnetismo, Universidad Rey Juan Carlos, Tulipán s/n, 28933 Móstoles Madrid, Spain; 2 Department of Physics, Lancaster University, Lancaster, LA1 4YB UK

## Abstract

In this work, we study the influence of orbital viscosity on the evolution of the order-parameter and texture in the B phase of superfluid ^3^He near a moving boundary. From the redistribution of thermal quasiparticles within the texture, we develop a model which confers a substantial effective mass on the interface, and provides a new mechanism for friction as the boundary moves. We have tested the model against existing data for the motion of an A-B interface whose motion was controlled by a magnetic field. The model allows us to make predictions for the behaviour in experimental situations which involve texture rearrangement arising from motion of the B-phase boundary.

## Introduction

Many systems^1–7^ condense into coherent states characterised by a “rigidity” to distortions in their wave function description^8^. Among such systems, superfluid ^3^He at very low temperature can condense in two phases, A and B. The bulk properties of the A and B phases are presently very well understood^9^. However close to boundaries, the topology of the order parameter changes and one might expect unconventional behaviour^10^.

In this context, the motivation for the present work arises from the very high dissipation observed when an A-B interface in superfluid ^3^He is spatially oscillated at very low temperatures ($T\sim 0.16{T}_{c}$), as reported previously^11^. In that work, the dissipation of a moving A-B interface was measured over a frequency range of 0.1 to 50 Hz. These very high dissipations have encouraged the formulation of the model presented here, and it is these measurements which provide the basis of comparison with the model.

To explain these results we have identified a new source of dissipation within the moving superfluid condensate that is generally taken to be frictionless. This mechanism should be relevant to all multiple-phase coherent condensates with non-trivial topologies. We show that there is an effective inertia and damping associated with the change in the topology of the orbital angular momentum. Using the model presented here, we are able to successfully test the results against previously unexplained experimental observations, to make quantitative predictions and further to propose additional experiments.

Superfluid ^3^He can be thought of as comprising three separate components: the mass superfluid, the spin superfluid and the orbital superfluid. The dynamics of the orbital superfluid is so strongly clamped by the normal fluid that it has been largely ignored in previous work. In zero magnetic field, the B phase of ^3^He is pseudo-isotropic, having no net spin or orbital angular momentum and an isotropic energy gap Δ_0_^12^. However, when exposed to a magnetic field the B-phase order parameter becomes distorted with the energy gap acquiring a minimum along the orbital anisotropy axis ***l***_*B*_. In a strong magnetic field (i.e. comparable to that required to stabilize the A-phase), the B-phase anisotropy becomes dominated by the Zeeman splitting resulting in a large density of thermal excitations occupying states along the ***l***_*B*_ axis. Any sudden change in the spatial orientation of ***l***_*B*_ will generate substantial dissipation as the thermal excitations must redistribute.

The redistribution of thermal excitations has two components. First, there is a dissipative component which can be related to the orbital viscosity, as discussed by Fisher and Suramlishvili^13^, and secondly, a reactive component which can be viewed as an effective mass, which we have discussed in an earlier paper^14^ in which we reported a very preliminary attempt at introducing orbital dynamic ideas specifically to explain the specific case of the behavior of a moving A-B interface. Since then we have realized that our initial treatment can be extended and is not only appropriate to the moving phase interface, but can be given much more general validity allowing it to be applied to any B-phase boundary, e.g. at a solid boundary to a wire surface or moving plate. This new extension of the applicability of these ideas is the subject of the current paper.

In general, the orbital textures bend near boundaries. At solid walls, for example, say in a cylindrical geometry, with the magnetic field parallel to the cylindrical axis, the energetically favorable spatial distribution of ***l***_*B*_ is the “flare-out” texture^15–17^. Here, ***l***_*B*_ lies parallel to the magnetic field in the bulk liquid far from the walls and bends to accommodate the condition that it must lie perpendicular to the side walls. The orbital axis ***l***_*B*_ also bends at an interface between the A and the B phases, stabilized by a magnetic field gradient (at low temperatures and pressures the critical field for the A-B phase transition is *B*_*c*_ = 340 mT). In the bulk B phase ***l***_*B*_ lies parallel to the magnetic field direction, but at the interface the energetically favoured orientation is that parallel to the interface^18^.

In this work we study the effect of the change of the orbital direction at the boundaries of the B-phase in magnetic fields due to the movement of these boundaries. As we are interested in the change of the tilt angle, taking as reference the direction of the magnetic field, this can be considered a nutation motion when described using Euler angles. We apply the results to describe the dynamical behavior of an oscillating A-B interface at very low temperatures and in high magnetic fields and also to provide predictions on what will happen when the B-phase boundaries move. The dissipation will depend on the orientation of the movement of the boundary with respect to the equilibrium texture configuration.

## Results

### The model

Following Schopohl^19^ and Ashida and Nagai^20^, the energy of a quasiparticle excitation with momentum *p* and spin $\sigma \hslash$ in a magnetic field is given by:

1 $${E}_{p,\sigma }=\sqrt{{({E}_{\parallel }(p)-\sigma \hslash {\tilde{\omega }}_{L})}^{2}+{({{\rm{\Delta }}}_{\perp }{\hat{p}}_{\perp })}^{2}}.$$

here ${E}_{\parallel }(p)={({\xi }^{2}+{{\rm{\Delta }}}_{\parallel }^{2}{\hat{p}}_{\parallel }^{2})}^{\frac{1}{2}}$ (with $\xi =(p-{p}_{F}){v}_{F}$) is the kinetic energy relative to the Fermi energy. ${{\rm{\Delta }}}_{\parallel }$ and Δ_⊥_ are the energy gaps parallel and perpendicular to ***l***_*B*_, ${\hat{p}}_{\parallel }$ and ${\hat{p}}_{\perp }$ are the parallel and perpendicular components of the quasiparticle momentum unit vector, *v*_*F*_ is the Fermi velocity and *p*_*F*_ is the Fermi momentum. The quasiparticle Zeeman energy, with Fermi-liquid corrections, is $\sigma \hslash {\tilde{\omega }}_{L}$ where *σ* = ±1/2. Since the Zeeman splitting dominates the anisotropy, the two gap components, ${{\rm{\Delta }}}_{\parallel }$ and Δ_⊥_, can be approximated by the zero field gap Δ_0_^20–22^. The minimum quasiparticle energy depends on the angle between the quasiparticle momentum and the ***l***_*B*_ axis. At temperatures well below *T*_*c*_ more or less all the excitations occupy the lowest energy states, with momenta centered around the ***l***_*B*_ axis. Thus any change in the local orientation of ***l***_*B*_ will alter the quasiparticle energies and the subsequent relaxation of the excitations back to equilibrium will proceed on a time scale *τ*. This provides the fundamental mechanism by which an effective mass and friction is imparted to moving boundaries.

A change in the orientation of ***l***_*B*_ by *δ****θ*** gives rise to a change in the quasiparticle energies of

2 $$\delta {E}_{p,\sigma }=\frac{\partial {E}_{p,\sigma }}{\partial {\boldsymbol{\theta }}}\cdot \delta {\boldsymbol{\theta }},$$

where ***θ*** is the angle between the magnetic field direction and the ***l***_*B*_ vector.

This produces a viscous torque^23,24^

3 $${{\boldsymbol{\Gamma }}}_{vis}=-\mu {{\boldsymbol{l}}}_{B}\times {\dot{{\boldsymbol{l}}}}_{B}=-\,\mu \dot{{\boldsymbol{\theta }}},$$

where *μ* is the orbital viscosity.

In the B phase at low temperatures, the orbital viscosity *μ* was previously shown to be^13^

4 $$\mu =\frac{\tau +i\omega {\tau }^{2}}{1+{(\omega \tau )}^{2}}[\frac{\pi }{6}N(0)\frac{{\rm{\Delta }}}{{k}_{B}T}\exp (-{\rm{\Delta }}/{k}_{B}T){(\hslash {\tilde{\omega }}_{L})}^{2}]$$

to first order in ${\tilde{\omega }}_{L}^{2}$. Here, *τ* is the quasiparticle relaxation time, *N*(0) is the normal density of states at the Fermi surface, and Δ we can take as approximately equal to the zero-field gap Δ_0_.

If we assume that the B-phase orbital texture responds instantaneously to any change in the position of the interface, then we may neglect any orbital dynamics and presume that ***l***_*B*_ always takes up its equilibrium orientation ***θ***(**r** − **r**_*I*_) relative to the boundary interface *r*_*I*_. This adiabatic approximation is ultimately justified as long as the characteristic time of the motion of the boundary is much greater than *τ*. Any fuller treatment in the future should also take into account the dynamics of the ***l***_*B*_ texture itself. Now let us consider a small change in the position of the boundary interface *δ***r**_*I*_. This will result in the orientation of the texture in the B phase adjusting by

5 $$\delta {\boldsymbol{\theta }}({\bf{r}})=\nabla {\boldsymbol{\theta }}\cdot \delta {{\bf{r}}}_{I}$$

meaning that

6 $$\dot{{\boldsymbol{\theta }}}=\nabla {\boldsymbol{\theta }}\cdot {\dot{{\bf{r}}}}_{I}.$$

Note that ▽***θ*** is a tensor of the second order.

The corresponding work done on the quasiparticle distribution can be written:

7 $$\delta W=-{\int }_{V}{{\boldsymbol{\Gamma }}}_{vis}\cdot \delta {\boldsymbol{\theta }}dV={\int }_{V}\mu \dot{{\boldsymbol{\theta }}}\cdot \delta {\boldsymbol{\theta }}dV$$

the integral being taken over the whole of the B-phase volume.

The corresponding effect on the boundary dynamics can be found by equating the work done by the viscous torque (7) to that done by the moving boundary. The force exerted per unit surface by the moving boundary, considering only the effect of the viscous torque, will have both a reactive (inertial) and dissipative (frictional) component, ${\bf{F}}={m}_{l}{\ddot{{\bf{r}}}}_{I}+{\gamma }_{l}{\dot{{\bf{r}}}}_{I}$. We can decompose the motion into the Fourier modes, so ${\ddot{{\bf{r}}}}_{I}=i\omega {\dot{{\bf{r}}}}_{I}$ and the work done on moving the interface by *δ***r**_*I*_ can be written as:

8 $$\delta W={\int }_{s}({\gamma }_{l}+i\omega {m}_{l}){\dot{{\bf{r}}}}_{I}\cdot \delta {{\bf{r}}}_{I}\,dS.$$

Setting this equal to the work done on the quasiparticles, Eq. (7), along with expressions (5) and (6) gives:

9 $${\int }_{s}({\gamma }_{l}+i\omega {m}_{l}){\dot{{\bf{r}}}}_{I}\cdot \delta {{\bf{r}}}_{I}dS=\mu {\int }_{V}(\nabla {\boldsymbol{\theta }}\cdot {\dot{{\bf{r}}}}_{I})\cdot (\nabla {\boldsymbol{\theta }}\cdot \delta {{\bf{r}}}_{I})dV.$$

Thus the real part of the orbital viscosity provides the friction coefficient *γ*_*l*_ and the imaginary part provides the effective mass *m*_*l*_. Note that this expression is valid for any angle between the surface normal and the angular momentum vector.

### Equilibrium texture parallel to the interface

**Figure 1:**
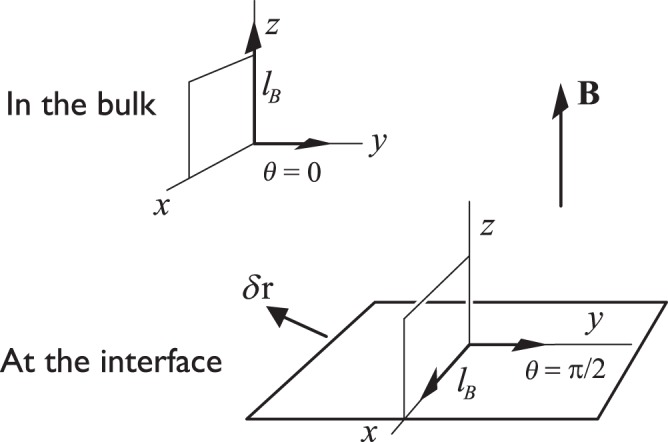
Sketch of the case discussed in the main text. The interface lies in the *xy* plane and we take the magnetic field **B** as pointing in the *z* direction. In the bulk, the ***l***_*B*_ texture lies parallel to the magnetic field **B** in the *z* direction. On approaching the interface, ***l***_*B*_ must rotate to become parallel to the interface, following the trajectory illustrated in Fig. 2. The vector *δ***r** denotes the translation of the interface in a random direction.

**Figure 2:**
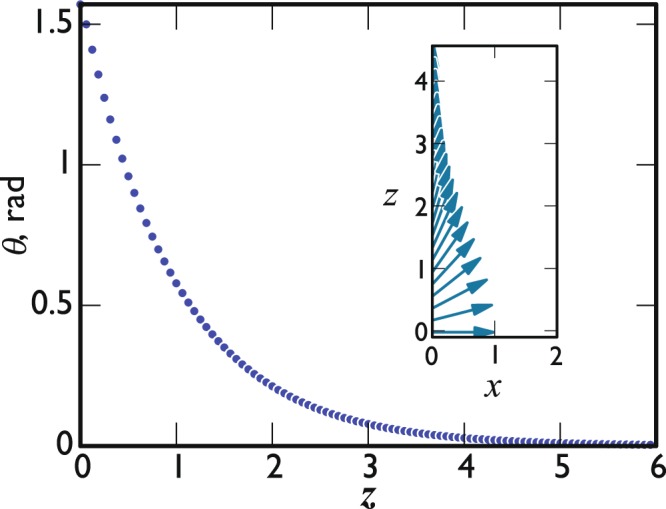
Numerical calculation of the ***l***_*B*_ texture near the A-B interface. *θ* is the angle between ***l***_*B*_ and the ***z*** axis. The points are a plot of (*π*/2)exp(−(*z* − *z*_*I*_)/*ξ*_*B*_) taking *z*_*I*_ at the origin. In the inset is plotted ***l***_*B*_ as a vector of unit length in arbitrary units, showing the change in the orientation. The ***x*** and ***z*** axes are in units of *ξ*_*B*_.

The expression of Eq. (9) allows us to make some predictions. Let us take the simplest case where the equilibrium texture configuration is parallel to the interface which is in the horizontal direction, and in the bulk the ***l***_*B*_ texture is vertical, in the direction of the magnetic field **B**. The situation is shown in Fig. 1. In this configuration ***l***_*B*_ must turn towards the horizontal as the interface is approached^18^. This rotation takes place over a distance of order *ξ*_*B*_. The corresponding texture has been calculated numerically by finding the configuration which minimizes the bending energy and the magnetic free energy simultaneously^24^. It is plotted in Fig. 2 where *θ* is the angle between ***l***_*B*_ and the vertical axis ***z***.

Thus, in this particular situation the texture ***θ***(*z*) is described well by

10 $${\theta }_{x}={\theta }_{z}=0,$$

11 $${\theta }_{y}=\frac{\pi }{2}\exp (-(z-{z}_{I})/{\xi }_{B}).$$

Now take as the displacement of the interface a translation in the ***x*** and ***z*** directions,

12 $$\delta {{\bf{r}}}_{I}=\delta r(\cos \,\varphi ,0,\,\sin \,\varphi ),$$

so

13 $${\dot{{\bf{r}}}}_{I}=\dot{r}(\cos \,\varphi ,0,\,\sin \,\varphi ).$$

Then using (9) and (11) we get

14 $${\int }_{s}({\gamma }_{l}+i\omega {m}_{l})dS=\mu {\int }_{V}{(\frac{\partial {\theta }_{y}}{\partial {z}_{I}})}^{2}{\sin }^{2}\varphi \,dV.$$

Thus, for *ϕ* = 0, which corresponds to a boundary displacement parallel to the interface, the effective mass and dissipation disappears, while the maximum will occur for a displacement in the normal direction. It must be noted that the presence of any defect in the texture on the boundary or a different equilibrium configuration of the texture will change this prediction, but nevertheless we expect that there should be preferred directions for the effective mass and the dissipation.

### The oscillating A-B interface

We can apply the model to an oscillating A-B phase interface. The dynamics of such an interface is particularly interesting at low temperatures. It has been proposed that the interface has an effective mass and there is dissipation due to pair-breaking and Andreev scattering when it moves^10^. Indeed at higher interface velocities the pair-breaking has analogies with Schwinger pair-creation and the Unruh effect in particle physics^25^. It was found experimentally^26^ that the friction values of a rapid freely-advancing interface in low magnetic fields were consistent with theoretical estimates based on the Andreev scattering of thermal quasiparticle excitations^27^. However, in subsequent measurements in Lancaster in high magnetic fields and much lower temperatures^28^ the observed friction was found to be orders of magnitude greater than the theoretical predictions^10,27,29^. The measurements were made on an interface that was stabilized by a field gradient and which could be driven into controlled oscillation using shaped magnetic field profiles, at much lower temperatures where pair-breaking was expected to dominate the dissipation^11^. Furthermore, the dissipation showed non-linear behavior that appeared to depend on the frequency of the oscillations of the interface. It was pointed out that the motion of an oscillating A-B interface in high magnetic fields and at low temperatures should be dominated by the orbital viscosity and by a significant effective mass arising from the redistribution of thermal quasiparticle excitations confined in the B-phase order parameter texture^14^. The change of the surrounding texture and energies of thermal quasiparticles should contribute to the effective mass of the interface and also produce dissipation arising from the orbital viscosity^13^.

The motion of the interface can be described by^27^

15 $$m{\ddot{{\bf{r}}}}_{I}+\gamma {\dot{{\bf{r}}}}_{I}={{\bf{n}}}_{AB}{\rm{\Delta }}{G}_{AB},$$

where **n**_*AB*_ the surface normal directed toward the B-phase, and ${\rm{\Delta }}{G}_{AB}=\mathrm{1/2}{\chi }_{AB}({B}_{c}^{2}-{B}^{2})$ the Gibbs energy difference per unit volume for the two phases with *χ*_*AB*_ being the difference in the magnetic susceptibilities of the A and B phases.

To simplify the calculations, we will again assume that the B-phase response to the changing position of the interface is instantaneous is and that the boundary remains flat during its motion. This last approximation is valid as long as the healing length *ξ*_*B*_ over which ***l***_*B*_ changes direction is smaller than the size of the boundary and the effect of the side walls in the form of a meniscus can be neglected. We note, that in the A-phase the preferred orientations of the orbital vector ***l***_*A*_ in the bulk and on the surface are the same, so the orbitropic effect does not manifest in the A-phase when the interface is moving along the direction of the magnetic field.

Under those conditions Eq. (9), after substituting *dV* = *Adz* with *A* the area of the interface, reads

16 $${\gamma }_{l}+i\omega {m}_{l}=\mu {\int }_{z}{(\frac{\partial {\theta }_{y}}{\partial {z}_{I}})}^{2}dz.$$

We can estimate the integral in Eq. (16) by assuming that the angle *θ* changes exponentially from *π*/2 to zero over the textural healing length *ξ*_*B*_, as given by Eq. (11). The integral of Eq. (16) then becomes:

17 $${\int }_{z}{(\frac{\partial {\theta }_{y}}{\partial {z}_{I}})}^{2}dz=\frac{{\pi }^{2}}{8{\xi }_{B}}=\frac{1.2337}{{\xi }_{B}}.$$

The numerical evaluation of this integral, based on the texture configuration which minimises the bending energy and the magnetic free energy simultaneously, gives 1.2353/*ξ*_*B*_ instead.

Substituting Eqs (4) and (17) in Eq. (16) gives the following estimates for the friction coefficient

18 $${\gamma }_{l}=\frac{\tau }{1+{(\omega \tau )}^{2}}[\frac{\pi }{6}N(0)\frac{{\rm{\Delta }}}{{k}_{B}T}\exp (\,-\,{\rm{\Delta }}/{k}_{B}T){(\hslash {\tilde{\omega }}_{L})}^{2}]\frac{{\pi }^{2}}{8{\xi }_{B}}$$

and the effective mass

19 $${m}_{l}=\frac{{\tau }^{2}}{1+{(\omega \tau )}^{2}}[\frac{\pi }{6}N\mathrm{(0)}\frac{{\rm{\Delta }}}{{k}_{B}T}\exp (\,-\,{\rm{\Delta }}/{k}_{B}T){(\hslash {\tilde{\omega }}_{L})}^{2}]\frac{{\pi }^{2}}{8{\xi }_{B}}.$$

So that the friction and mass parameters of Eq. (15) become *m* = *m*_*l*_ and *γ* = *γ*_0_ + *γ*_*l*_. The contribution *γ*_0_ comes from Andreev scattering and pair breaking^10,27,29^, or any dynamical friction with the walls.

**Figure 3:**
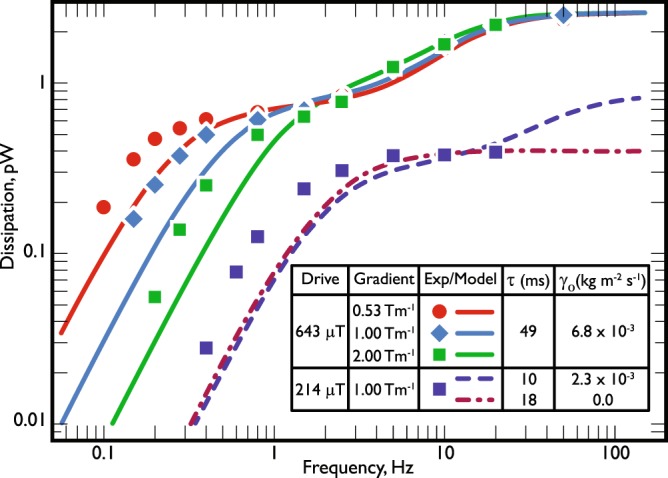
Dissipation of the oscillating A-B interface versus frequency. The points show measurements at a field oscillation amplitude of *B*_*ac*_ = 0.643 mT for three different field gradients 2.00, 1.00 and 0.53 T/m, and *B*_*ac*_ = 0.214 mT for the gradient 1.00 T/m. The lines are fits to the model (see text). The inset table summarizes the results of the fits. The temperature varies from 150 to 160 *μ*K over a single set of measurements, rising slowly as the dissipation increases with increasing frequency. Measurements are only possible up to around 100 Hz, beyond which the dissipation becomes too high to stabilise the temperature.

Let us now test the results on real experimental data, those plotted in Fig. 3. The experiment in which these data were taken is described in short in ref.^11^ and in more detail by Arrayás *et al*.^14^. Briefly, the measurements were performed on a sample of superfluid ^3^He contained in a sapphire tube, closed at one end and at the other end connected through an orifice to the inner cell of a Lancaster-style nuclear cooling stage. The volume inside the tube acts as a quasiparticle black-body radiator. Any dissipation in the superfluid contained in the tube generates an excess of quasiparticle excitations which are lost through the orifice to the bulk superfluid outside. From the temperature (≡ quasiparticle density) measured inside and outside the radiator volume we can deduce the energy flux flowing out of the orifice, which provides a very accurate measure of the dissipation in the radiator volume. (These devices are remarkable sensitive being able to detect energy inputs on the 10^−16^ W level.) The sapphire tube has internal diameter 4.3 mm and length 44 mm. The experiments were carried out at a pressure of 0 bar. A solenoid stack was used to create a shaped magnetic field profile to stabilize the A-phase in the bottom of the tube in fields above the transition field *B*_*c*_ = 340 mT^28,30,31^, whilst maintaining the top of the tube in the low field B-phase. Once the A-B interface was established across the tube, a small additional alternating field was applied to oscillate the interface vertically over a range of frequencies.

To evaluate *γ*_*l*_ and *m*_*l*_ for the experimental data in Fig. 3 we took the following values: $\hslash {\tilde{\omega }}_{L}=0.67{{\rm{\Delta }}}_{0}$, from^20–22^; Δ_0_ = 1.76*k*_*B*_*T*_*c*_ with *T*_*c*_ = 0.929 mK at 0 bar pressure, from^32^; *N*(0) = 1.0 × 10^51^ J^−1^m^−3^, from^32,33^; and *T* = 155 *μ*K (noting that this is an average temperature, and the actual temperature during the measurements varied from *T* = 150 *μ*K to *T* = 160 *μ*K as the dissipation increased from low to high frequencies). We assume *ξ*_*B*_ = 0.1 mm, using some estimations for fields close to *B*_*c*_^17,18,34,35^.

The only unknown parameters in our model are the quasiparticle relaxation time *τ* and the frequency independent dissipation term *γ*_0_, which is an additive term to *γ*_*l*_ in (18). The model predictions are shown together with the experimental data in Fig. 3. We have found that two values of *τ* on the order of tens of milliseconds are needed to fit the experimental data, depending on the magnitude of the driving magnetic field. The value for *τ* calculated for the *uniform* texture and for the given experimental conditions is only a few milliseconds^36^. With the texture bending, the quasiparticles may become trapped within the texture, which would increase their relaxation time. In the case of the dissipation parameter *γ*_0_, two different values are needed as well to successfully fit the measured dependence. The values of *γ*_0_ roughly scale as the amplitudes of the driving field (their ratio being about 3). For the set of experimental data in the case of the 214*μ*T drive, we have also considered the fitting with no extra constant dissipation. The lack of experimental data at higher frequencies prevents us from drawing further conclusions. We remark that texture bending could be a function of the driving amplitude and remains to be further investigated.

## Discussion

The data we fit are shown in Fig. 3 which are those reported in refs^11,14^. To model the double-plateau profile of the data as a driven damped harmonic oscillator, the effective mass and the dissipative coefficient must be frequency dependent, as shown by Eqs (18) and (19). As can be seen the model reproduces the principal features of the measured data. It follows the double-plateau form reasonably well. Unfortunately, the data taken at 214 *μ*T do not extend to high enough temperature for the upper plateau to be reached at this drive amplitude.

We should emphasise that the double-plateau profile is certainly real and not associated with any saturation effect from heating by the moving boundary. In other similar measurements we certainly see no temperature gradients in the cells which would imply heating at this point.

The data are of great interest, and especially because the dissipation is so much higher than would be naively expected without taking into account the response of the orbital moment which we model here, which can be seen from the way the ideas of how to deal with the data have evolved. In ref.^11^ we followed the then current wisdom that these effects were the result of Andreev reflection, but a simple linear friction fit on that basis simply did not reproduce the observed data. In ref.^14^ we had understood that the effect arose from orbital precession of some sort and managed to achieve a more realistic fit, albeit for a very restricted version of the interface. The current model now reproduces the features of the system rather well and is completely general for all configurations of the interface. When the original measurements were made, the high dissipation turned out to be a nuisance as we had no idea what we were actually seeing. Now that we have a clearer understanding of the contributing processes, this would be a good time to revisit the experiment but now with a clearer view of the contribution of the orbital evolution. In future experiments the orbitropic effect can also be studied in other, less complicated situations. For example, in a magnetic field in the vicinity of a thin superconducting wire carrying alternating current. The magnetic field generated by this current will reorient the texture in ^3^He-B, thus causing dissipation (the magnetic field on the surface of a 0.1 mm thick wire carrying 1A is 4 mT and the field gradient is 80 T/m).

We would also like to comment on the results of a recent paper concerning the motion of the A-B interface^37^. The paper predicts a very peculiar form of a magnetic wave that can be excited at the interface: as the two superfluid phases have different magnetic susceptibilities, the phase transition between them is accompanied by a change in magnetisation. The author estimates that the effective inertia of such a wave is not large enough for the wave to be excited in a typical experiment. However, the orbitropic effect will endow the wave with a high value of the effective mass: at the resonant frequency of the wave (*f*_res_ = 220 Hz for typical experimental conditions) our calculation (19) gives *m*_*l*_ = 8.5 × 10^−7^ kg/m^2^ which is about 100 times greater than that predicted in^37^. As a result, the dissipation rate of 35 s^−1^ is small compared to 2*πf*_res_ and makes the observation of the magnetic phase wave more realistic.

In conclusion, in this work we have considered a new dissipation mechanism based on the redistribution of thermal quasiparticles in the orbital texture. We have investigated its effect at the boundaries of the B-phase, and studied the consequences of the change of the nutation angle of the orbital vector ***l***_*B*_. We have estimated the effective mass and dissipation associated with this motion, assuming adiabatic approximation of the influence of the orbital dynamics on the boundary dynamics. The main result is expressed in Eq. (9). Further, we have applied the theory developed to the dynamics of an oscillating A-B interface. The results are in a reasonable agreement with the experimental data and explain the increase in dissipation for frequencies above 2 Hz which could not be explained by existing theories.

## Data Availability

All data used in this paper are available at 10.17635/lancaster/researchdata/239, including descriptions of the data sets.
